# Objective interictal electrophysiology biomarkers optimize prediction of epilepsy surgery outcome

**DOI:** 10.1093/braincomms/fcab042

**Published:** 2021-03-14

**Authors:** Naoto Kuroda, Masaki Sonoda, Makoto Miyakoshi, Hiroki Nariai, Jeong-Won Jeong, Hirotaka Motoi, Aimee F Luat, Sandeep Sood, Eishi Asano

**Affiliations:** 1 Department of Paediatrics, Children’s Hospital of Michigan, Detroit Medical Centre, Wayne State University, Detroit, MI 48201, USA; 2 Department of Epileptology, Tohoku University Graduate School of Medicine, Sendai 9808575, Japan; 3 Department of Neurosurgery, Yokohama City University, Yokohama 2360004, Japan; 4 Swartz Centre for Computational Neuroscience, Institute for Neural Computation, University of California San Diego, La Jolla, CA 92093, USA; 5 Division of Paediatric Neurology, Department of Paediatrics, UCLA Mattel Children’s Hospital, David Geffen School of Medicine, Los Angeles, CA 90095, USA; 6 Department of Neurology, Children’s Hospital of Michigan, Detroit Medical Centre, Wayne State University, Detroit, MI 48201, USA; 7 Department of Paediatrics, Yokohama City University Medical Centre, Yokohama 2320024, Japan; 8 Department of Neurosurgery, Children’s Hospital of Michigan, Detroit Medical Centre, Wayne State University, Detroit, MI 48201, USA

**Keywords:** invasive recording, electrocorticography (ECoG), high-frequency oscillation (HFO), modulation index (MI), phase-amplitude coupling (PAC)

## Abstract

Researchers have looked for rapidly- and objectively-measurable electrophysiology biomarkers that accurately localize the epileptogenic zone. Promising candidates include interictal high-frequency oscillation and phase-amplitude coupling. Investigators have independently created the toolboxes that compute the high-frequency oscillation rate and the severity of phase-amplitude coupling. This study of 135 patients determined what toolboxes and analytic approaches would optimally classify patients achieving post-operative seizure control. Four different detector toolboxes computed the rate of high-frequency oscillation at ≥80 Hz at intracranial EEG channels. Another toolbox calculated the modulation index reflecting the strength of phase-amplitude coupling between high-frequency oscillation and slow-wave at 3–4 Hz. We defined the completeness of resection of interictally-abnormal regions as the subtraction of high-frequency oscillation rate (or modulation index) averaged across all preserved sites from that averaged across all resected sites. We computed the outcome classification accuracy of the logistic regression-based standard model considering clinical, ictal intracranial EEG and neuroimaging variables alone. We then determined how well the incorporation of high-frequency oscillation/modulation index would improve the standard model mentioned above. To assess the anatomical variability across non-epileptic sites, we generated the normative atlas of detector-specific high-frequency oscillation and modulation index. Each atlas allowed us to compute the statistical deviation of high-frequency oscillation/modulation index from the non-epileptic mean. We determined whether the model accuracy would be improved by incorporating absolute or normalized high-frequency oscillation/modulation index as a biomarker assessing interictally-abnormal regions. We finally determined whether the model accuracy would be improved by selectively incorporating high-frequency oscillation verified to have high-frequency oscillatory components unattributable to a high-pass filtering effect. Ninety-five patients achieved successful seizure control, defined as International League against Epilepsy class 1 outcome. Multivariate logistic regression analysis demonstrated that complete resection of interictally-abnormal regions additively increased the chance of success. The model accuracy was further improved by incorporating z-score normalized high-frequency oscillation/modulation index or selective incorporation of verified high-frequency oscillation. The standard model had a classification accuracy of 0.75. Incorporation of normalized high-frequency oscillation/modulation index or verified high-frequency oscillation improved the classification accuracy up to 0.82. These outcome prediction models survived the cross-validation process and demonstrated an agreement between the model-based likelihood of success and the observed success on an individual basis. Interictal high-frequency oscillation and modulation index had a comparably additive utility in epilepsy presurgical evaluation. Our empirical data support the theoretical notion that the prediction of post-operative seizure outcomes can be optimized with the consideration of both interictal and ictal abnormalities.

## Teaser

Kuroda et al. report the results of the computational analysis of intracranial EEG data from a cohort of 135 patients with drug-resistant focal epilepsy. Both interictal HFO and phase-amplitude coupling measures were found to improve the prediction of post-operative seizure outcome.

## Introduction

Complete resection of the epileptogenic zone is necessary to achieve successful seizure control in drug-resistant focal epilepsy.^1^ Clinicians at tertiary epilepsy centres across the world frequently rely on the seizure-onset zone (SOZ) defined on intracranial EEG (iEEG) and an MRI lesion to localize the epileptogenic zone.^2^^,^^3^ To optimize the diagnostic accuracy, investigators look for rapidly- and objectively-measurable electrophysiology biomarkers. We previously hypothesized that the rate of interictal spike discharges on iEEG would provide additive information to localize the epileptogenic zone and effectively improve the prediction of post-operative seizure outcomes. Previous iEEG studies indeed demonstrated that the SOZ was associated with increased spiking rates.^4^^,^^5^ However, our prospective iEEG study failed to prove that considering spike rates improves the seizure outcome classification model-based on the standard information, including clinical, SOZ and MRI data.^6^ Thus, we have looked for an alternative electrophysiology biomarker.

The promising biomarkers include interictal high-frequency oscillation (HFO), episodic and spontaneous iEEG signals at ≥80 Hz, augmenting in amplitude and standing out from the baseline.^7^^,^^8^ Investigators provide the open-source detectors, which automatically detect HFO events according to the own definition of HFO.^9–13^ The SOZ was reported to show higher HFO rates than the non-SOZ, whereas the effect size differed between studies.^13–16^ Investigators, who assessed the seizure outcome in relation to the extent of resection, further supported the utility of interictal HFO in epilepsy presurgical evaluation.^17^ A prospective iEEG study of 52 patients found a correlation between the removal of HFO-generating regions and seizure outcomes at the group level.^18^ In the present study, four open-source detectors computed the HFO rate at iEEG electrode sites.^9–12^ We investigated whether the incorporation of HFO rates would improve the standard outcome classification model considering the clinical, SOZ and MRI data achievable by the completion of resective surgery. We determined whether HFO rates quantified by different detectors would commonly enhance the accuracy of the outcome classification model.

The present study likewise assessed the diagnostic utility of phase-amplitude coupling between interictal HFO and slow-wave, rated by modulation index (MI).^19^^,^^20^ Since the present study has chosen to quantify the magnitude of high-frequency amplitude ≥80 Hz stereotypically coupled with subsequent slow-wave 3–4 Hz, MI effectively reflects the severity of spike-and-wave discharges.^21^^,^^22^ Two epilepsy centres independently reported that the phase-amplitude coupling was elevated at the SOZ; thereby, the coupling with slow-wave at 3–4 Hz best differentiated the SOZ from non-SOZ.^23^^,^^24^ We determined whether the standard outcome classification model would be improved by incorporating MI more than consideration of HFO, or *vice versa*.

The present study followed the recommendation to consider the anatomical variability in the HFO rate across non-epileptic sites in surgical planning.^25^^,^^26^ We generated the normative atlas of detector-specific HFO and MI; thus, each atlas allowed us to compute the statistical deviation (z-score) of HFO/MI from the normative mean using the concept of statistical parametric mapping.^27^ We determined whether the post-operative seizure outcome would be classified better by incorporating absolute or z-score normalized HFO/MI (referred to as zHFO/zMI below).

The present study finally determined whether investigators must selectively use HFO events verified to have distinct high-frequency oscillatory components unattributable to the results from a high-pass filtering effect on a very sharply-contoured transient.^28–30^ It remains to be determined whether such verified HFO (vHFO) would allow investigators to predict post-operative seizure outcomes more accurately. We determined whether the post-operative seizure outcome would be classified better by incorporating vHFO as defined by each of the four different detectors.

## Materials and methods

### Patients

We studied a consecutive series of 135 patients with drug-resistant focal epilepsy (Table 1) who underwent resective surgery between January 2007 and May 2018 at Detroit Medical Centre in Detroit, USA. The inclusion criteria included extraoperative iEEG recording with a sampling rate of 1000 Hz.^11^^,^^31^ The exclusion criteria included (i) diagnosis of bilateral multifocal epilepsy purely based on the Phase-1 non-invasive presurgical evaluation,^32^ (ii) callosotomy, hemispherotomy, or hemispherectomy,^33^^,^^34^ (iii) major brain malformations making the central or lateral sulcus unidentifiable,^35^ (iv) malignant brain tumour suspected on preoperative MRI,^31^ (v) post-operative follow‐up shorter than 1 year,^36^ (vi) age at surgery was <4 years^37^ and (vii) history of previous resective epilepsy surgery.^27^ The institutional review board at Wayne State University has approved the protocol. We obtained written informed consent from patients or the guardians of paediatric patients.

**Table 1:** Patient profile

Patients profile	
**Total number of patients**	**135**
Mean age	13.1 years old (range: 4–44)
Sex	68 males (50.4%)
Daily seizures	45 patients (33.3%)
Number of AEDs	
One AED	40 patients (29.6%)
Two AEDs	60 patients (44.4%)
Three AEDs	34 patients (25.2%)
Four AEDs	None (0%)
Five AEDs	1 patient (0.7%)
Left-hemispheric epilepsy	71 patients (52.6%)
Lesion visible on MRI	79 patients (58.5%)
Habitual seizures captured during iEEG recording	117 patients (86.7%)
Incomplete resection of SOZ	17 patients (12.6%)
Necessity to resect extra-temporal region	85 patients (63.0%)
Mean size of resection (%)	16.2% (range: 0.6–91.6)
Mean number of analysed electrodes per patient	108.2 electrodes (range: 32–152)
ILAE class 1 outcome	95 patients (70.4%)

AEDs = antiepileptic drugs taken immediately before the electrode placement; iEEG = intracranial EEG; SOZ = seizure-onset zone; ILAE = International League Against Epilepsy.

### iEEG

We placed platinum subdural electrodes for the SOZ localization.^6^^,^^31^^,^^35^ Subdural electrodes remain utilized in many paediatric epilepsy centres across the countries.^38–42^ Surface electrooculography and electromyography electrodes determined the onset of seizure-related symptoms and sleep staging.^31^^,^^43^ We recorded video-iEEG signals for 2–7 days with an amplifier band-pass of 0.016–300 Hz and performed analysis using common average reference.^43^^,^^44^ We discontinued antiepileptic drugs (AEDs) and resumed them following the localization of the SOZ.^6^ We excluded artifactual channels from further analyses.^44^ Thus, a total of 14 604 electrode channels were available for analysis.

### MRI

Preoperative 3 T MRI data, including T1-weighted three-dimensional spoiled gradient-echo volumetric scan and fluid‐attenuated inversion recovery scan, were reviewed by an experienced neuroradiologist.^31^^,^^32^ Implanted subdural electrodes were co-registered with a three‐dimensional surface image.^35^^,^^45^ Using the FreeSurfer scripts (http://surfer.nmr.mgh.harvard.edu. Accessed 22 March 2021), we created the averaged surface image to which all electrode locations were spatially normalized.^27^^,^^35^^,^^37^ The averaged surface image functioned as the template for the normative HFO/MI atlas.

### Surgery

Our previous studies described the principle to determine the extent of cortical resection.^6^^,^^31^ We intended to remove the SOZ and a neighbouring MRI lesion, if any, while maximally preserving the eloquent cortex. None of the clinicians or investigators were aware of quantitative HFO/MI analysis results before the resective surgery.

Using the intraoperative photographs immediately before dural closure, we prospectively declared whether the SOZ was removed entirely.^31^ The FreeSurfer scripts computed the extent of resection (i.e. percentage of the hemisphere).^31^ Post-operative MRI cannot function as a preoperative predictor. Using the Spearman’s rank test, our ancillary analysis assessed the concordance between the resection sizes estimated by intraoperative photographs and post-operative MRI in 89 patients whose post-operative MRI data were available to us (Supplementary Fig. 1).

### Computation of the rate of HFO

We used MATLAB R2019a (Mathworks, Natick, MA) for the following HFO analyses. The visual assessment initially identified the earliest, artifact-free, 5-min slow-wave sleep >2 h apart from ictal events^31^; then, the RIPPLELAB (https://github.com/BSP-Uniandes/RIPPLELAB/. Accessed 22 March 2021)^13^ quantified the rate of HFO events at each electrode site. The RIPPLELAB incorporates four distinct HFO detection algorithms as follows: (i) Short Time Energy (STE) method,^9^ (ii) Short Line Length (SLL) method,^10^ (iii) Hilbert (HIL) method^11^ and (iv) Montreal Neurological Institute (MNI) method.^12^ We used the default settings as incorporated in the RIPPLELAB^13^ (Supplementary Fig. 2A–D) and each HFO detection algorithm computed the rates of HFO≥80 Hz, HFO≥150 Hz and HFO≥250 Hz. Each original article describes a given detector’s principle in detail.^9–12^ In short, the STE method-defined an HFO event as an iEEG segment showing successive root mean square values greater than 5 SDs above the overall root mean square mean, including more than 6 peaks >3 SDs on the band-pass filtered iEEG.^9^ The SLL method-defined an HFO as a segment showing SLL-based amplitude augmentation greater than the 97.5th percentile of the empirical cumulative distribution function computed on band-pass filtered iEEG signals.^10^^,^^13^ The HIL method-defined an HFO as a segment showing HIL transform-based envelope augmentation greater than five SDs.^11^ The MNI method-defined an HFO as a segment showing root mean square-based energy above the 99.9999th percentile compared to the baseline if such a baseline period was considered to be present.^12^ If the baseline was absent, the MNI method treated a segment showing the ≥95th percentile of the cumulative distribution function as computed from the 1 min band-passed iEEG.

Below, for example, STE_>__*f*__Hz_ denotes the STE method-defined HFO in the frequency range at *f*-300 Hz; thereby, *f* was 80, 150, or 250.

### Computation of MI

All iEEG data points during the 5 min mentioned above were HIL transformed by the EEGLAB Toolbox winPACT (https://sccn.ucsd.edu/wiki/WinPACT. Accessed 22 March 2021).^20^ The toolbox automatically computed the MI quantifying the strength of coupling between HFO amplitude and the instantaneous phase of slow-wave_3__–__4 Hz_ (Supplementary Fig. 2E).^27^^,^^31^ Below, MI_>__*f*__Hz_ denotes the coupling between the amplitude of HFO_*f*__–__300 Hz_ and the phase of slow-wave_3__–__4 Hz_; thereby, *f* was 80, 150, or 250.

### Post-operative seizure outcome

Post-operative seizure outcome was classified at the last follow-up according to the International League Against Epilepsy (ILAE) classification.^36^ The Class-1 was treated as a success.^27^^,^^31^

### Normative atlas

**Figure 1:**
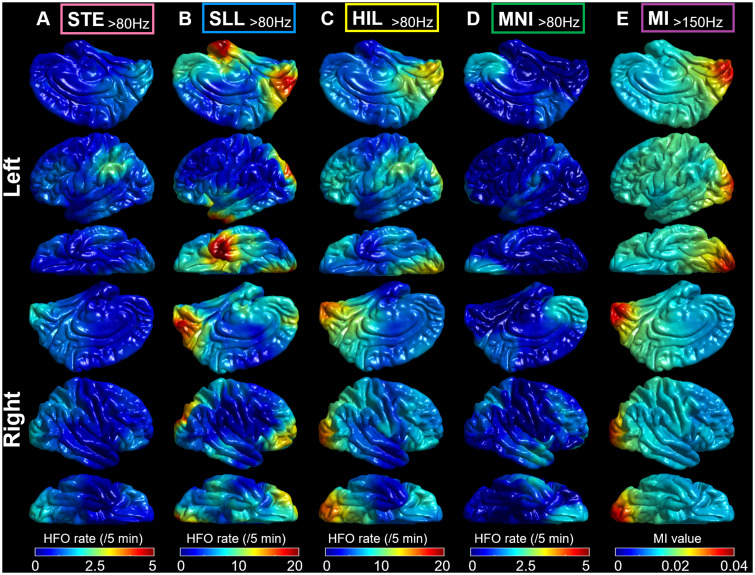
**Normative atlas of HFO and MI.** (**A–D**) Each normative atlas demonstrates the spatial characteristics of the rate of HFO_>80 Hz_ defined by each of the four different detectors. The mean HFO rate across 60 closest non-epileptic sites is presented. Note that each atlas has excluded outlier non-epileptic sites showing HFO rates greater than ten standard deviations higher than the mean. (**A**) STE detector.^9^ (**B**) SLL detector.^10^ (**C**) HIL detector.^11^ (**D**) MNI detector.^12^ (**E**) The normative atlas of MI_>150 Hz_ shows the characteristics of phase-amplitude coupling between HFO_>150 Hz_ and slow wave_3–4 Hz_.

We generated the normative atlases of HFO defined by four detectors mentioned above. We used the method used to create the normative MI atlas based on the 2477 non-epileptic electrode sites sampled from 47 patients.^27^ Non-epileptic sites were defined as those outside the SOZ, cortical lesions and spiking zones.^25^ We computed the mean and standard deviation of the HFO rate at each surface model mesh vertex based on the 60 closest non-epileptic sites. Figure 1 visualizes the mean non-epileptic HFO rate and MI at the whole-brain level.

### Statistical analysis

We used SPSS v25 (IBM, Armonk, NY) and considered a two-sided *P*-value of 0.05 as significant. We determined how accurately the standard model incorporating the following 10 variables would classify patients achieving a surgical success (i.e. ILAE class 1 outcome). The predictor variables included (i) age, (ii) sex, (iii) presence of daily seizures, (iv) number of oral AEDs taken immediately before the intracranial electrode placement (reflecting the severity of seizure burden),^46^ (v) affected hemisphere, (vi) presence of an MRI lesion,^47^ (vii) whether iEEG recording captured habitual seizure events, (viii) whether the SOZ was completely removed, (ix) the necessity of extra-temporal lobe resection and (x) size of resection. We computed the *R*^2^ to assess the fitness of the standard model in outcome classification. The receiver-operating characteristics (ROC) analysis determined the model accuracy, as rated by the area under the ROC curve (AUC).

We determined how well incorporating the detector-specific HFO measures would improve the standard model’s accuracy in seizure outcome classification. We computed the accuracy of a given HFO model as follows. Each detector-specific HFO model incorporated Subtraction-HFO in addition to the ten variables mentioned above. Subtraction-HFO was defined as the subtraction of the HFO rate averaged across all preserved sites from that averaged across all resected sites. Subtraction-HFO effectively quantified the completeness of the resection of interictal HFO-generating regions. Higher Subtraction-HFO inferred more complete resection of the area showing a focal increase of HFO rate.

With the ROC analysis, we likewise assessed how accurately the MI model, incorporating Subtraction-MI in addition to the 10 variables mentioned above, would classify patients who achieved surgical success. Subtraction-MI was defined as the subtraction of the MI averaged across all preserved sites from that averaged across all resected sites.

**Figure 2:**
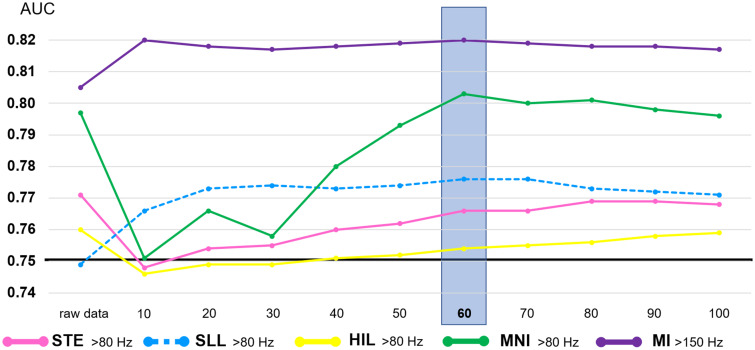
**Effect of the number of non-epileptic electrode sites included for computation of the normative mean/standard deviation.** We delineated how the classification accuracy of the zHFO and zMI models would be altered by the analytic approach (i.e. the number of non-epileptic sites to be included for computation of the normative mean and standard deviation). The classification accuracy of zHFO and zMI models was stable when ≥60 closest non-epileptic sites were included. Conversely, inclusion of as small as 10 non-epileptic sites resulted in a worsening of the classification accuracy by some zHFO models. This observation can be explained by the notion that these HFO detectors were designed to effectively avoid detecting HFO_>80 Hz_ at most non-epileptic sites and that the computation of standard deviation of HFO rate was not tenable with 10 closest sites. The one-way ANOVA test indicated that the accuracy of outcome classification differed among these five models (*P* < 0.001). The post-hoc paired *t*-test indicated that the zMI model had higher classification accuracy compared to all zHFO models (Bonferroni-corrected *P* < 0.05 on paired *t*-test). The standard model had a classification accuracy of 0.75 as indicated by a thick line. AUC: Area under the curve on the ROCs analysis.

The subsequent analysis assessed the generalizability of the notion that investigators should consider the anatomical variability of HFO/MI across non-epileptic sites.^25–27^ Specifically, each electrode site was assigned a z-score normalized HFO rate (zHFO) and MI (zMI), as computed using the normative mean and standard deviation across 60 closest non-epileptic channels (Fig. 1).^27^ The ROC analyses likewise determined the outcome classification accuracy of a given zHFO model and zMI model (i.e. that incorporating Subtraction-zHFO/zMI instead of Subtraction-HFO/MI). For interested readers, further analysis determined how many closest non-epileptic sites (range: 10–100) should be included for computing a z-score normalized HFO/MI that would result in an optimal classification of patients achieving surgical success (Fig. 2).

**Figure 3:**
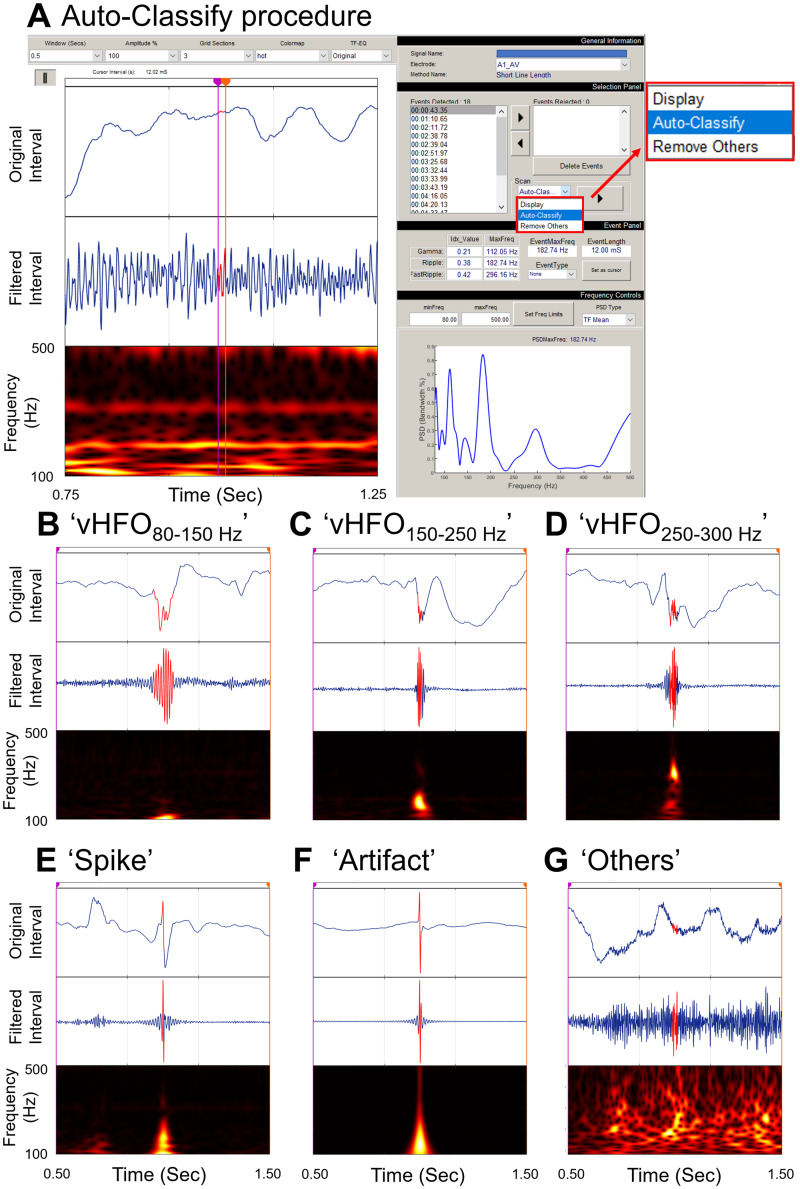
**Verification process using the RIPPLELAB.** (**A**)The snapshot of HFO verification process using the auto-classify function implemented in the RIPPLELAB. Based on the spectral characteristics, this function allowed us to label each detected event of HFO as either **B** ‘vHFO_80–150 Hz_’, ‘vHFO_150–250 Hz_’ in **C**, ‘vHFO_250–300 Hz_’ in **D**, ‘Spike’ in **E**, ‘Artifact’ in **F** or ‘Others’ in **G**. vHFO_80–150 Hz_ denotes an HFO event verified to have distinct high-frequency oscillatory components at 80–150 Hz unattributable to the results from an 80-Hz high-pass filtering on a very sharply-contoured transient. (**B–G**)Top image: Unfiltered intracranial EEG (iEEG) trace. Middle image: Filtered iEEG trace. Bottom image: Time-frequency plot.

The subsequent analysis determined whether investigators must selectively use HFO events verified to have distinct high-frequency oscillatory components unattributable to the results from a high-pass filtering effect on a very sharply-contoured transient.^28–30^ Such distinct high-frequency oscillatory components are reflected by a blob-like power increase on the time-frequency plot (Fig. 3B–D).^28^ The auto-classify function, implemented in the RIPPLELAB^13^, verified whether each detected HFO event was unattributable to the filtering (Fig. 3). Based on the spectral characteristics, this function labelled each detected event as ‘vHFO_80__–__150Hz_’, ‘vHFO_150__–__250Hz_’, ‘vHFO_250__–__300Hz_’, ‘Spike’, ‘Artifact’, or ‘Others’. Thereby, vHFO_*f*__Hz_ denotes HFO verified to have a distinct high-frequency oscillatory component at *f* Hz. This verification procedure was extremely time-consuming (up to three investigator hours/patient). We computed the rates of vHFO events; thereby, vHFO_>80 Hz_ included vHFO_80__–__150 Hz_, vHFO_150__–__250 Hz_ and vHFO_250__–__300 Hz_. Likewise, vHFO_>150 Hz_ included vHFO_150__–__250 Hz_ and vHFO_250__–__300 Hz_. We determined the outcome classification accuracy of a given vHFO model (i.e. that incorporating Subtraction-vHFO instead of Subtraction-HFO).

Leave-one-out analysis^27^ cross-validated the standard outcome prediction model and each of those incorporating interictal electrophysiology biomarkers mentioned above. We estimated the probability of surgical success of each new patient based on the multivariate logistic regression model incorporating all variables derived from the remaining 134 patients. The AUC of ROC curves determined how much the leave-one-out cross-validation altered each model’s performance.

We finally measured the computational time (seconds/channel) required to quantify the HFO rate and MI. We used a Windows 10 laptop computer with Intel Core i7 at 2.7–2.9 GHz processor, 16.0 GB random-access memory and a 64-bit operating system (NEC Corporation, Tokyo, Japan).

### Data and code availability

All data and code used in this study are available upon request to the corresponding author. We are pleased to re-analyse the data and provide the results based on readers’ specific suggestions to improve our understanding of the neurobiology of epilepsy.

## Results

### Post-operative seizure outcome

ILAE class 1, 2, 3, 4, 5 and 6 outcomes were noted in 95, 2, 15, 14, 9 and 0 patients (mean follow‐up: 65.4 months [SD: 38.2; range: 12–130]).

### Resection size

The resection size estimated by the intraoperative photo was concordant with that by the post-operative MRI (Spearman rho: 0.95; *P* < 0.001; Supplementary Fig. 1).

### Utility of the standard model in outcome classification

**Figure 4:**
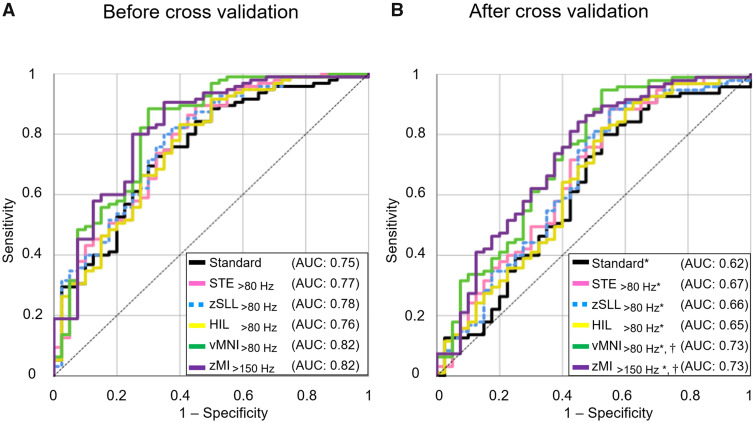
**The accuracy of models incorporating interictal electrophysiology biomarkers.** (**A**) A given ROCs curve delineates the accuracy of seizure outcome classification of a given model. Black line: Standard model. Pink line: STE_>80 Hz_ model. Green line: zSLL_>80 Hz_model incorporating SLL_>80 Hz_ (i.e. z-score normalized SLL_>80 Hz_). Yellow line: HIL_>80 Hz_ model. Orange line: vMNI_>80 Hz_model incorporating vMNI_>80 Hz_ (i.e. MNI_>80 Hz_ verified to have distinct high-frequency oscillatory components unattributable to the effect of high-pass filtering). Purple line: zMI_>150 Hz_ model. The areas under the ROC curves were greater than 0.5 (Bonferroni-corrected *P* <0.001 on Mann-Whitney U test). Both HFO- and MI-based models improved the outcome classification by the standard model (accuracy: 0.75 → up to 0.82).(**B**) The ROC curves with a leave-one-out approach used. Both HFO- and MI-based models improved the outcome prediction based on the standard model (accuracy: 0.62 → up to 0.73). Comparison of 134 ‘sensitivity × specificity’ values consisting of each ROC plot^31^ determined whether the size of AUC for a given HFO/MI-based model differed from the chance level (i.e. 0.5) and that of the standard model. We found that all five HFO/MI-based models had AUC larger than 0.5 (*, Bonferroni-corrected *P* < 0.001 on *t*-test). vMNI_>80 Hz_ and zMI_>150 Hz_ models had AUC larger than that of the standard model (†, Bonferroni-corrected *P* < 0.001 on *t*-test).

The standard model classified the post-operative seizure outcome with significance (*R*^2^: 0.23, *P* = 0.008). Incomplete SOZ resection (odds ratio [OR]: 0.15 [95%CI: 0.05–0.51]) and a larger number of AEDs (OR: 0.56 [0.33–0.97]) were independently associated with a smaller chance of success (Supplementary Table 1). The accuracy of outcome classification was 0.75, as rated by the AUC of the ROC curve (Fig. 4A).

### Normative atlas

Figure 1 presents the spatial distributions of detector-specific HFO_>80 Hz_ and MI_>150 Hz_ within non-epileptic sites. Non-epileptic occipital lobe sites were associated with higher HFO_>80 Hz_ rates compared to other lobes (*P* < 0.001 on the linear mixed model analysis). The SLL (mean rate across all non-epileptic sites: 5.1/5 min) and HIL (5.9/5 min) detectors detected HFO_>80 Hz_ more than the STE (0.9/5 min) and MNI (0.7/5 min) (*P* < 0.001 on the linear mixed model analysis).

### Values of HFO, zHFO and vHFO

HFO_>80 Hz_ improved the outcome classification accuracy of the standard model better than HFO_>150 Hz_ and HFO_>250Hz_. Thus, we described the performance of HFO_>80 Hz_ in the Results Section below.

The classification accuracy of the STE_>80 Hz_, SLL_>80 Hz_, HIL_>80 Hz_ and MNI_>80 Hz_ models was 0.77, 0.75, 0.76 and 0.80 (Supplementary Fig. 3A–D). Higher Subtraction-STE_>80 Hz_, Subtraction-HIL_>80 Hz_ and Subtraction-MNI_>80 Hz_ were independently associated with a greater chance of success (OR: 1.23 [95%CI: 1.03–1.46]; OR: 1.05 [1.01–1.10]; OR: 1.18 [1.04–1.33]) (Table 2).

**Table 2:** Accuracy of the HFO, zHFO, vHFO, MI and zMI models in outcome classification

Model	HFO model			zHFO model			vHFO model		
	*P*	OR	95% CI	*P*	OR	95% CI	*P*	OR	95% CI
STE_>80 Hz_	**0.02**	1.23	1.03–1.46	0.08	1.27	––	**0.03**	1.52	1.05–2.20
STE_>150 Hz_	0.11	2.32	––	NA	NA	NA	0.23	2.75	––
STE_>250 Hz_	0.45	4.71 × 10^–15^	––	NA	NA	NA	>0.99	8.58 × 10^–178^	––
SLL_>80 Hz_	0.08	1.02	––	**0.01**	1.15	1.03–1.29	**0.03**	1.10	1.01–1.19
SLL_>150 Hz_	0.78	1.01	––	0.28	1.03	––	0.18	1.16	––
SLL_>250 Hz_	0.20	2.02	––	0.74	1.00	––	0.83	1.27	––
HIL_>80 Hz_	**0.02**	1.05	1.01–1.10	0.09	1.28	––	0.09	1.33	––
HIL_>150 Hz_	0.17	1.30	––	0.78	1.00	––	0.25	2.63	––
HIL_>250 Hz_	0.33	5.39 × 10^–35^	––	NA	NA	NA	>0.99	3.00 × 10^–266^	––
MNI_>80 Hz_	**0.008**	1.18	1.04–1.33	**0.002**	1.22	1.07–1.38	**0.04**	10.80	1.16–1.01 × 10^2^
MNI_>150 Hz_	0.08	1.60	––	0.78	1.00	––	**0.005**	1.82 × 10^5^	36.51–9.07 × 10^8^
MNI_>250 Hz_	0.38	1.58	––	0.76	1.00	––	0.42	2.03 × 10^2^	––

Model	MI model			zMI model		
	*P*	OR	95% C.I.	*P*	OR	95% C.I.
MI_>80 Hz_	**0.01**	4.75 × 10^2^	4.01–5.64 × 10^4^	**0.01**	1.20	1.04–1.39			
MI_>150 Hz_	**0.02**	7.23 × 10^11^	1.23 × 10^2^–4.26 × 10^21^	**0.01**	1.28	1.05–1.56			
MI_>250 Hz_	**0.02**	4.76 × 10^41^	1.25 × 10^6^–1.81 × 10^77^	**0.02**	1.23	1.03–1.46			

All toolboxes could generate a model that accurately classified the post-operative seizure outcome with significance (*P*-value < 0.05). CI = confidence interval; HFO = high-frequency oscillation; MI = modulation index; OR = odds ratio; *P* = *P*-value. zHFO/zMI model: That incorporating z-score normalized HFO/MI instead of absolute HFO/MI. vHFO model: That incorporating HFO verified to have high-frequency oscillatory components unattributable to a high-pass filtering effect. NA (not available): we were unable to perform the z-score normalization for STE_>150 Hz_, STE_>250 Hz_ and HIL_>250 Hz_ due to the lack of detected events in non-epileptic regions. *P* < 0.05 indicates significance in **bold** typeface.

**Figure 5:**
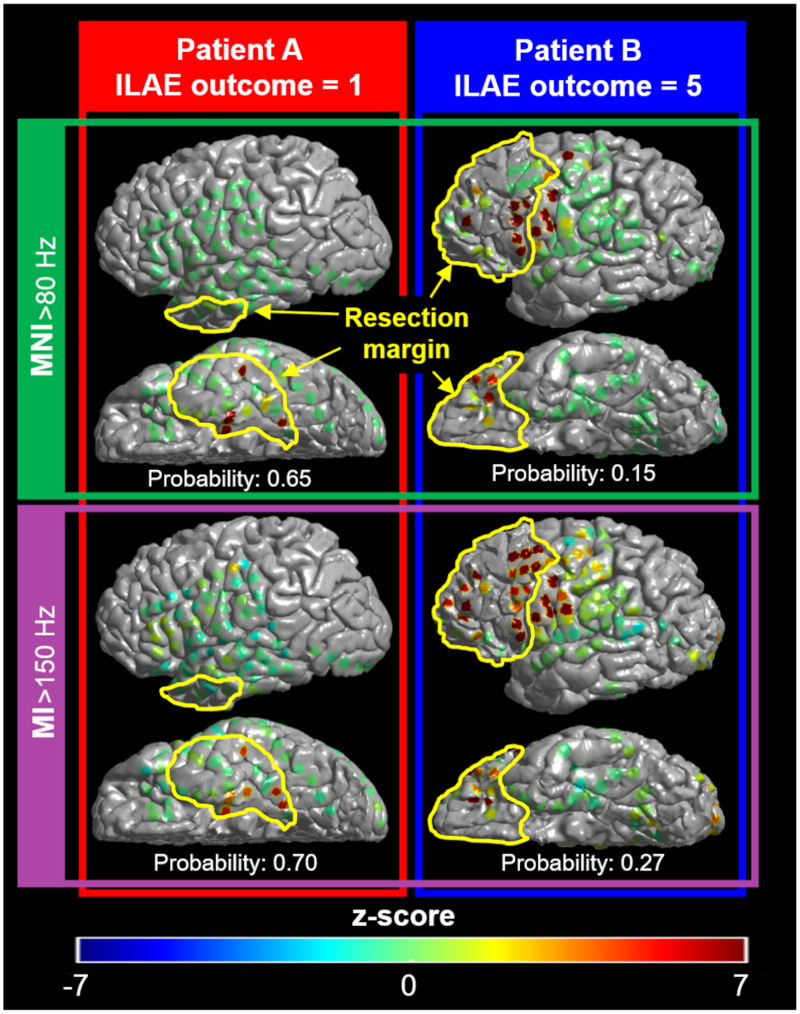
**Relationship between interictal electrophysiology biomarker, resection margin and post-operative seizure outcome.** The colour of each electrode site reflects the severity of interictal abnormality rated by the HFO rate as well as the MI. The yellow lines denote the resection margin in a given patient. Both MNI_>80 Hz_ and zMNI_>80 Hz_ models, incorporating interictal HFO rates defined by the MNI detector,^12^ suggested that only patient A had a high probability to achieve surgical success. The MI_>150 Hz_ and zMI_>150 Hz_ models, incorporating the phase-amplitude coupling rated by MI,^20^ made a similar outcome prediction. Indeed, Patient A achieved the ILAE class 1 outcome,^36^ whereas Patient B had a ILAE class 5 outcome.

The classification accuracy of the zSTE_>80 Hz_, zSLL_>80 Hz_, zHIL_>80 Hz_ and zMNI_>80 Hz_ models was 0.77, 0.78, 0.75 and 0.80 (Supplementary Fig. 4A–D). Higher Subtraction-zSLL_>80 Hz_ and Subtraction-zMNI_>80 Hz_ were associated with a greater chance of success (OR: 1.15 [95%CI: 1.03–1.29]; OR: 1.22 [1.07–1.38]) (Table 2 and Fig. 5).

The classification accuracy of the vSTE_>80 Hz_, vSLL_>80 Hz_, vHIL_>80 Hz_ and vMNI_>80 Hz_ models was 0.77, 0.77, 0.75 and 0.82 (Supplementary Fig. 5). Higher Subtraction-vSTE_>80 Hz_, Subtraction-vSLL_>80 Hz_ and Subtraction-vMNI_>80 Hz_ were associated with a greater chance of success (OR: 1.52 [95%CI: 1.05–2.20]; OR: 1.10 [1.01–1.19]; OR: 10.80 [1.16–1.01 × 10^2^]) (Table 2).

### Outcome classification using the MI and zMI models

MI_>150 Hz_ improved the standard model better than MI_>80 Hz_ and MI_>250 Hz_. Thus, we described the performance of MI_>150 Hz_ in the Results section below. The MI_>150 Hz_ and zMI_>150 Hz_ models improved the standard model, and the outcome classification accuracy was 0.81 and 0.82 (Supplementary Figs. 3E and 4E). Higher Subtraction-MI_>150 Hz_ (OR: 7.23 × 10^11^ [95%CI: 1.23 × 10^2^–4.26 × 10^21^]) and Subtraction-zMI_>150 Hz_ (OR: 1.28 [1.05–1.56]) were independently associated with a greater chance of success (Table 2 and Fig. 5).

### Cross-validation

We cross-validated STE_>80 Hz_, zSLL_>80 Hz_, HIL_>80 Hz_, vMNI_>80 Hz_ and zMI_>150 Hz_ models because a given analytic approach optimized the outcome classification performance. Following the leave-one-out cross-validation, the accuracy of outcome prediction of the standard, STE_>80 Hz_, zSLL_>80 Hz_, HIL_>80 Hz_, vMNI_>80 Hz_ and zMI_>150 Hz_ model was 0.62, 0.67, 0.66, 0.65, 0.73 and 0.73, respectively (Bonferroni-corrected *P* < 0.001 [significantly greater than 0.5]; Fig. 4B). Furthermore, the accuracies of outcome prediction of the vMNI_>80 Hz_ and zMI_>150 Hz_ models were higher than that of the standard model (Bonferroni-corrected *P* < 0.001; Fig. 4B).

**Figure 6:**
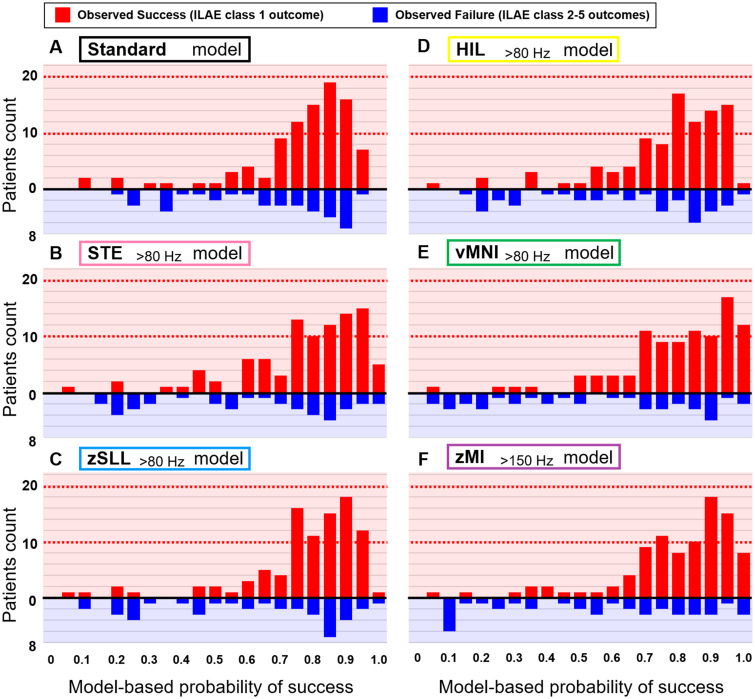
**Agreement between the model-based prediction and the observed frequency of surgical success.** X-axis: Model-based probability of surgical success for a given patient; each model was cross-validated by the leave-one-out procedure. Red bar: Number of patients achieving the ILAE class 1 outcome. Blue bar: The class 2 outcome or worse. (**A**) The standard model anticipated that 55 patients would achieve surgical success with a probability of greater than 0.8. Thereby, 42 out of these 55 patients (76%) indeed achieved surgical success. The standard model anticipated that 5 patients would achieve surgical success with a probability of smaller than 0.2. However, four out of these five patients (80%) still achieved surgical success. (**B**) The STE_>80 Hz_ model. (**C**) The zSLL_>80 Hz_ model. (**D**) The HIL_>80 Hz_ model. (**E**) The vMNI_>80 Hz_ model. (**F**) The zMI_>150 Hz_ model anticipated that 61 patients would achieve surgical success with a probability of greater than 0.8. Indeed, 51 out of these 61 patients (84%) achieved surgical success. The zMI_>150 Hz_ model anticipated that 10 patients would achieve surgical success with a probability of smaller than 0.2. Indeed, only two out of these 10 patients (20%) achieved surgical success.

Figure 6 demonstrates a generous agreement between the model-based likelihood of success and the observed frequency of success on an individual basis. Supplementary Fig. 6 presents the relationship between the model-based outcome prediction and the observed ILAE outcome scale. The Spearman rank test indicated that increased model-based probability of success was associated with better post-operative seizure outcome (Bonferroni-corrected *P* < 0.05; Supplementary Fig. 6).

### Computational time

The computational time was 3.7 s/channel for the STE-based HFO detection (95% CI: 3.55–3.85), 4.0 for the SLL (3.83–4.17), 4.2 for the HIL (4.04–4.36) and 89.3 for the MNI (84.73–93.87) and 1.2 for the MI quantification (1.15–1.25).

## Discussion

### Summary

Our overall results support the hypothesis that interictal HFO/MI measures improve the accuracy of the outcome classification based on the measurements available for standard care, including clinical, neuroimaging and ictal iEEG information. The effect size of improvement in the outcome classification accuracy (from 0.75 up to 0.82) was comparable between HFO and MI. Our study supports the conceptual notion that the combined consideration of both interictal and ictal abnormalities would optimally localize the zone generating focal seizures.^1^

### Pros and cons of each of the tested biomarkers

We want to congratulate all investigators involved in the development of HFO detectors and MI quantification toolbox. None of the interictal electrophysiology biomarkers was superior to others in all aspects, including precision, accuracy, speediness and interpretability. MI_>150 Hz_ and zMI_>150 Hz_ models improved the outcome classification accuracy from 0.75 to 0.81 and 0.82, respectively. MI, a continuous variable, might have characterized the spatial gradience in the severity of spike-and-wave discharges during a short interictal period better than the HFO rate, which is a discrete variable. ‘MI of 1’ may be more difficult to intuitively understand its meaning than ‘1 HFO event per minute’. We previously reported that iEEG epochs showing MI of 1 were characterized by abundant spike-and-wave discharges.^31^ By definition, a higher magnitude of high-frequency amplitude_≥80 Hz_ stereotypically coupled with slow-wave_3__–__4 Hz_ effectively increases the MI.^19^^,^^20^ A recent iEEG study of 11 patients recently reported that the HFO amplitude classified the SOZ more accurately than the HFO rate.^48^

MNI_>80 Hz_, zMNI_>80 Hz_ and vMNI_>80 Hz_ models had a classification accuracy of 0.80, 0.80 and 0.82. The rate of MNI_>80 Hz_ within the non-epileptic areas was smaller than those of SLL_>80 Hz_ and HIL_>80 Hz_. Perhaps, the MNI detector is designed to be agnostic to continuous forms of HFO,^12^ often taking place in the non-epileptic occipital areas.^23^ Thus, some might perceive that MNI_>80 Hz_ would be easy to incorporate in clinical practice. The computational time of the MNI detector was longer than those of the other HFO or MI toolboxes. One may have to use a high-speed computer to complete the MNI-based HFO analysis to allow clinicians to make decisions in a real-time manner, particularly in the operation room.

SLL_>80 Hz_, zSLL_>80 Hz_ and vSLL_>80 Hz_ had the outcome classification accuracy of 0.75, 0.78 and 0.77. The utility of SLL_>80 Hz_ became significant following the z-score normalization procedure, possibly because the default setting was designed to detect HFO events inclusively and broadly.^13^

STE_>80 Hz_, zSTE_>80 Hz_ and vSTE_>80 Hz_ had the outcome classification accuracy of 0.77, 0.77 and 0.77. HIL_>80 Hz_, zHIL_>80 Hz_ and vHIL_>80 Hz_ had the classification accuracy of 0.76, 0.75 and 0.75. Neither STE_>80 Hz_ nor HIL_>80 Hz_ benefitted from the z-score normalization or verification process. Both STE and HIL detectors required a short computational time; thus, they can promptly provide the results.

### Methodological considerations

We analysed the earliest 5-min slow-wave sleep epoch free from artefacts based on the visual assessment. This criterion is feasible for clinicians who would promptly need to decide the resection margin and adheres to the recommendation made in the guidelines of the use of HFO detectors.^49^ Selection of a longer iEEG epoch for analysis (e.g. days)^16^ could have optimized the normative HFO atlas and also detected more HFO events, particularly HFO_>250 Hz_ suggested to be a more specific but less sensitive biomarker than HFO_>80 Hz_.^7^^,^^8^^,^^50^^=^^52^ We are afraid that such an analysis of prolonged periods might increase the complexity of HFO/MI analysis. One should consider the effects of additional factors, including electromyographic artefacts such as those related to saccadic eye movements during REM sleep and wakefulness,^53^ post-ictal states,^54^ tissue swelling-related iEEG changes^55^ and after-discharges associated with electrical stimulation mapping.^56^ One must perform visual screening to identify and exclude artifactual channels during the tested interictal iEEG period to allow any of these toolboxes to quantify HFO or MI reliably. For the clinical translation of HFO/MI analysis, a given team must complete the iEEG signal analysis before the resective surgery, which typically occurs within a few days after the intracranial electrode placement.

The effect of the applied sampling rate (1000 Hz) on HFO detection is expected to be modest.^57^ Previous iEEG studies with a sampling rate of 2000 Hz indicated that the spectral frequency of neocortical HFO events mostly ranged below 300 Hz.^50–52^ The vast majority of our patients were children, and only six had hippocampal sclerosis on MRI. iEEG recorded with a 1000-Hz sampling rate would require half of the data storage space needed for 2000-Hz.

What is the generalizability of our single-centre study? We computed the MI alone to assess the severity of phase-amplitude coupling between HFO and slow-wave, whereas other computation methods have been reported elsewhere.^58–60^ With the default setting to detect HFO events, we generated the normative atlases, which demonstrated a noticeable variability in the spatial distribution of HFO rates across different toolboxes (Fig. 1). Some may raise the question of whether a specific toolbox should be recommended for iEEG clinical practice. Others may want to test different HFO detection settings further to optimize the outcome prediction method using our or their own dataset. We currently plan to determine our HFO/MI models’ generalizability using iEEG data acquired at different institutions and also to assess their utility in children younger than 4 years of age. External validation would be necessary before making a strong recommendation for the standard practice.

The utility of HFO/MI on intraoperative iEEG or stereo-EEG recording remains uncertain. A study reported that the presence of intraoperative HFO_>250 Hz_ immediately after the resection was associated with a higher risk of failure; simultaneously, these investigators recommended that one should be aware of physiological HFO_>250 Hz_ generated by eloquent cortices.^17^ Stereo-EEG allows us to sample the bottom of a sulcus which may include the epileptogenic zone, but its electrode density is low in a horizontal direction. We plan to develop the normative atlases of HFO/MI measured under the general anaesthesia and on stereo-EEG to determine the generalizability of the HFO/MI’s utility.

## Conclusion

Interictal HFO and MI had a comparably additive utility in epilepsy presurgical evaluation. Our empirical data support the theoretical notion that the prediction of postoperative seizure outcomes can be optimized with the consideration of both interictal and ictal abnormalities.

## Supplementary material

Supplementary material is available at *Brain Communications* online.

## Supplementary Material

fcab042_Supplementary_Data

## Abbreviations

AEDs =: antiepileptic drugs
AUC =: area under the curve
HFO =: high-frequency oscillation
HIL =: Hilbert
iEEG =: intracranial EEG
ILAE =: International League against epilepsy
MI =: modulation index
MNI =: Montreal Neurological Institute
RMS =: root mean square
ROC =: receiver-operating characteristics
SDs =: standard deviations
SLL =: short line length
SOZ =: seizure-onset zone
STE =: short time energy
