# Supplementary material for: Objective interictal electrophysiology biomarkers optimize prediction of epilepsy surgery outcome
Source: Brain Commun. 2021 Mar 14;3(2):fcab042. doi: 10.1093/braincomms/fcab042 (PMC8088817; doi:10.1093/braincomms/fcab042)
Supplement: fcab042_Supplementary_Data [file fcab042_supplementary_data.docx]

**Objective interictal electrophysiology biomarkers optimize prediction of epilepsy surgery outcome**

Naoto Kuroda, MD ^$^; Masaki Sonoda, MD, PhD ^$^; Makoto Miyakoshi, PhD ;

Hiroki Nariai, MD, PhD, MS ; Jeong-Won Jeong, PhD ; Hirotaka Motoi, MD, PhD ;

Aimee F. Luat, MD ; Sandeep Sood, MD ; Eishi Asano, MD, PhD, MS (CRDSA)*

$: Equal contribution.

*Corresponding author: [easano@med.wayne.edu](mailto:easano@med.wayne.edu)

**Supplementary Figure 1: Concordance between the resection sizes estimated by intraoperative photographs and postoperative MRI.**

**Supplementary Figure 2: Settings to quantify the HFO rate and MI.**

**Supplementary Figure 3: Classification accuracy of HFO and MI models.**

**Supplementary Figure 4: Classification accuracy of zHFO and zMI models.**

**Supplementary Figure 5: Classification accuracy of vHFO models.**

**Supplementary Figure 6: Correlation between model-based success probability and the ILAE outcome scale.**

**Supplementary Table 1: Outcome classification by the standard model.**


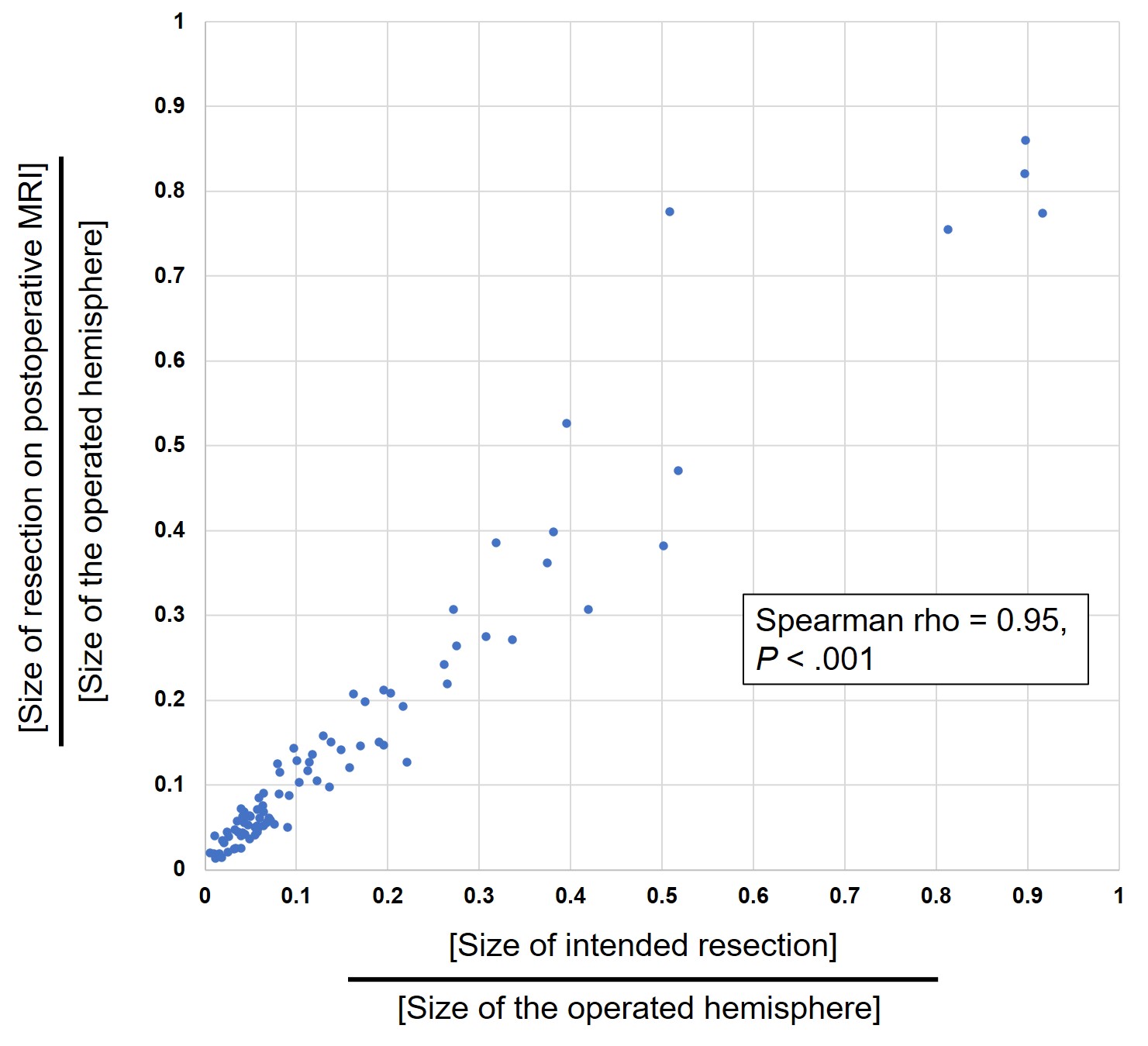


**Supplementary Figure 1: Concordance between the resection sizes estimated by intraoperative photographs and postoperative MRI.**

X-axis: Resection size (%) estimated by the intraoperative photographs. Y-axis: Resection size estimated by the postoperative MRI. The Spearman rank test demonstrated that the photo- and MRI-based resections sizes were tightly correlated (Spearman rho = 0.95; *P* < .001).

**
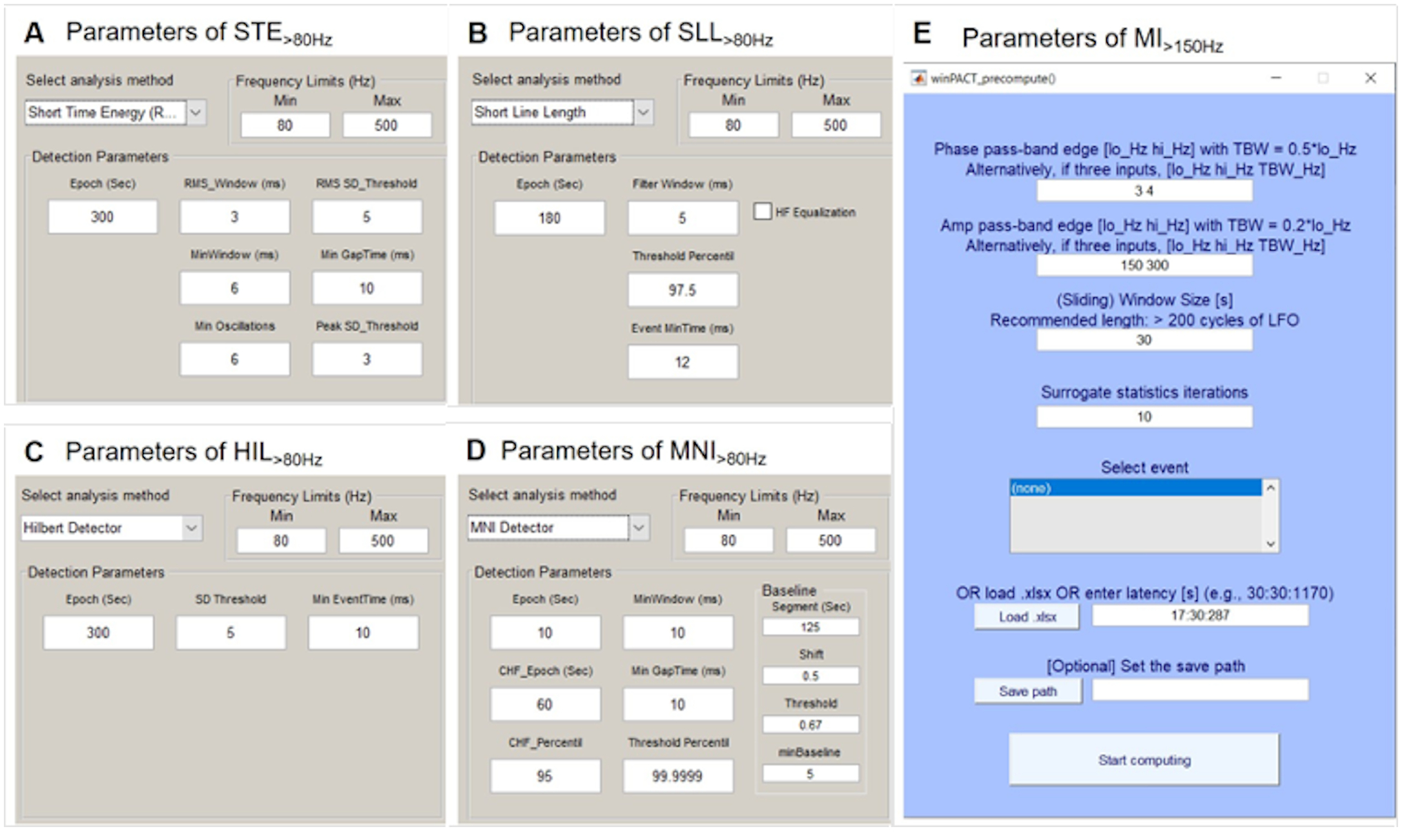
**

**Supplementary Figure 2: Settings to quantify the HFO rate and MI.**

We employed the RIPPLELAB default settings. (A-D) Each snapshot demonstrates the default setting for detection of events of high-frequency oscillation (HFO) at >80 Hz on the RIPPLELAB Toolbox (<https://github.com/BSP-Uniandes/RIPPLELAB/>). (A) The Short Time Energy (STE) method. (B) The Short Line Length (SLL) method. (C) The Hilbert (HIL) method. (D) The Montreal Neurological Institute (MNI) method. (E) The snapshot presents the setting to compute the modulation index (MI), quantifying the severity of phase-amplitude coupling between HFO_>150 Hz_ and slow wave_3-4 Hz_, on the EEGLAB winPACT Toolbox (<https://sccn.ucsd.edu/wiki/WinPACT>).

**
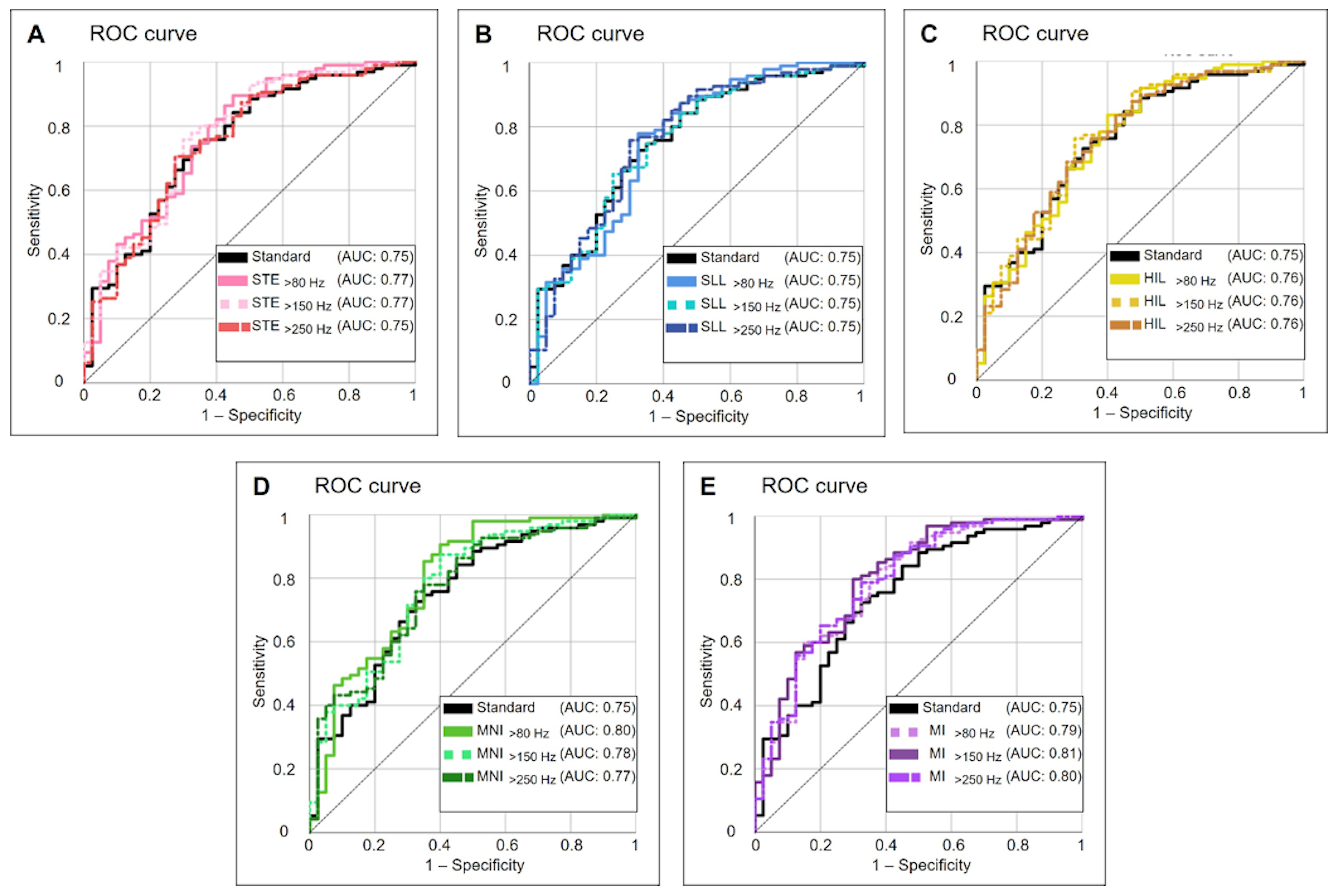
**

**Supplementary Figure 3: Classification accuracy of HFO and MI models.**

The receiver-operating characteristics (ROC) curves to assess the accuracy of seizure outcome classification. (A) Short Time Energy (STE) models. (B) Short Line Length (SLL) models. (C) Hilbert (HIL) models. (D) Montreal Neurological Institute (MNI) models. (E) Modulation index (MI) models.

**
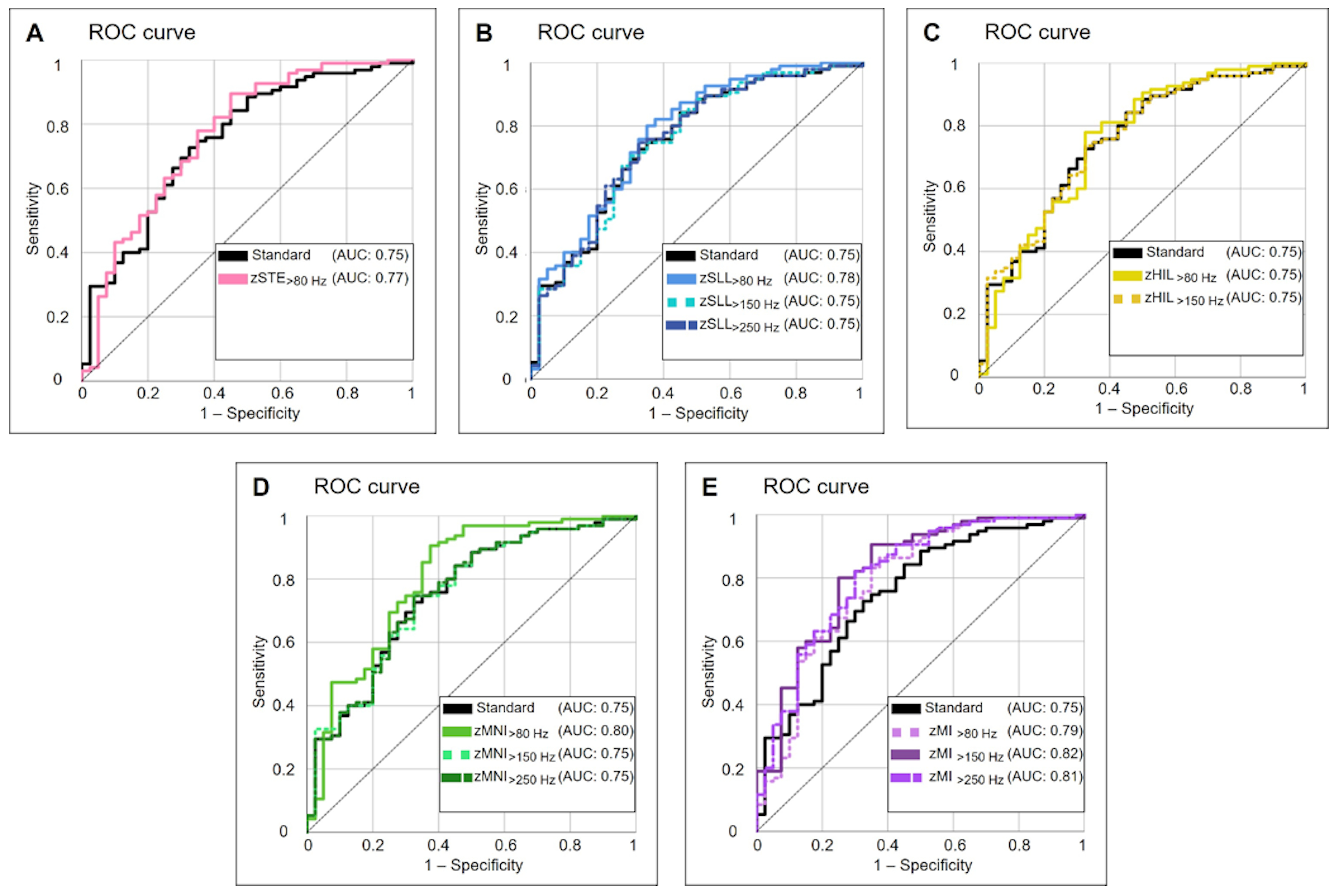
**

**Supplementary Figure 4: Classification accuracy of zHFO and zMI models.**

The receiver-operating characteristics (ROC) curves to assess the accuracy of seizure outcome classification by zHFO and zMI models. Each model incorporates the rate of z-score normalized HFO (zHFO) or z-score MI (zMI). (A) Short Time Energy (STE) models. (B) Short Line Length (SLL) models. (C) Hilbert (HIL) models. (D) Montreal Neurological Institute (MNI) models. (E) Modulation index (MI) models. We were unable to perform the z-score normalization for STE_>150 Hz_, STE_>250 Hz_, and HIL_>250 Hz_ due to the lack of detected events in non-epileptic regions.

**
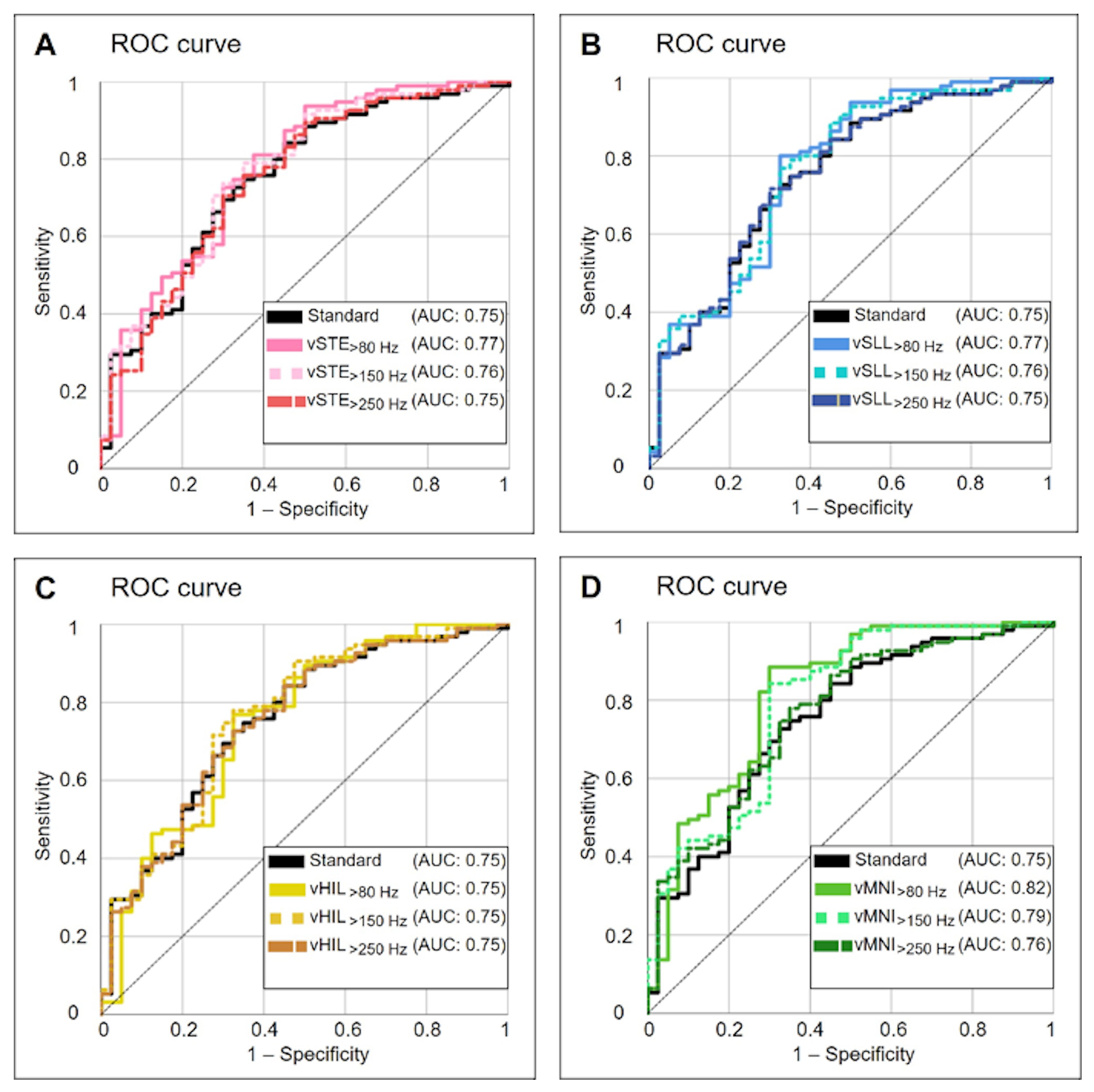
**

**Supplementary Figure 5: Classification accuracy of vHFO models.**

The receiver-operating characteristics (ROC) curves to assess the accuracy of seizure outcome classification by vHFO models. Each model incorporates the rate of HFO verified to have high-frequency oscillatory components unattributable to the effect of high-pass filtering (vHFO). (A) Short Time Energy (STE) models. (B) Short Line Length (SLL) models. (C) Hilbert (HIL) models. (D) Montreal Neurological Institute (MNI) models.


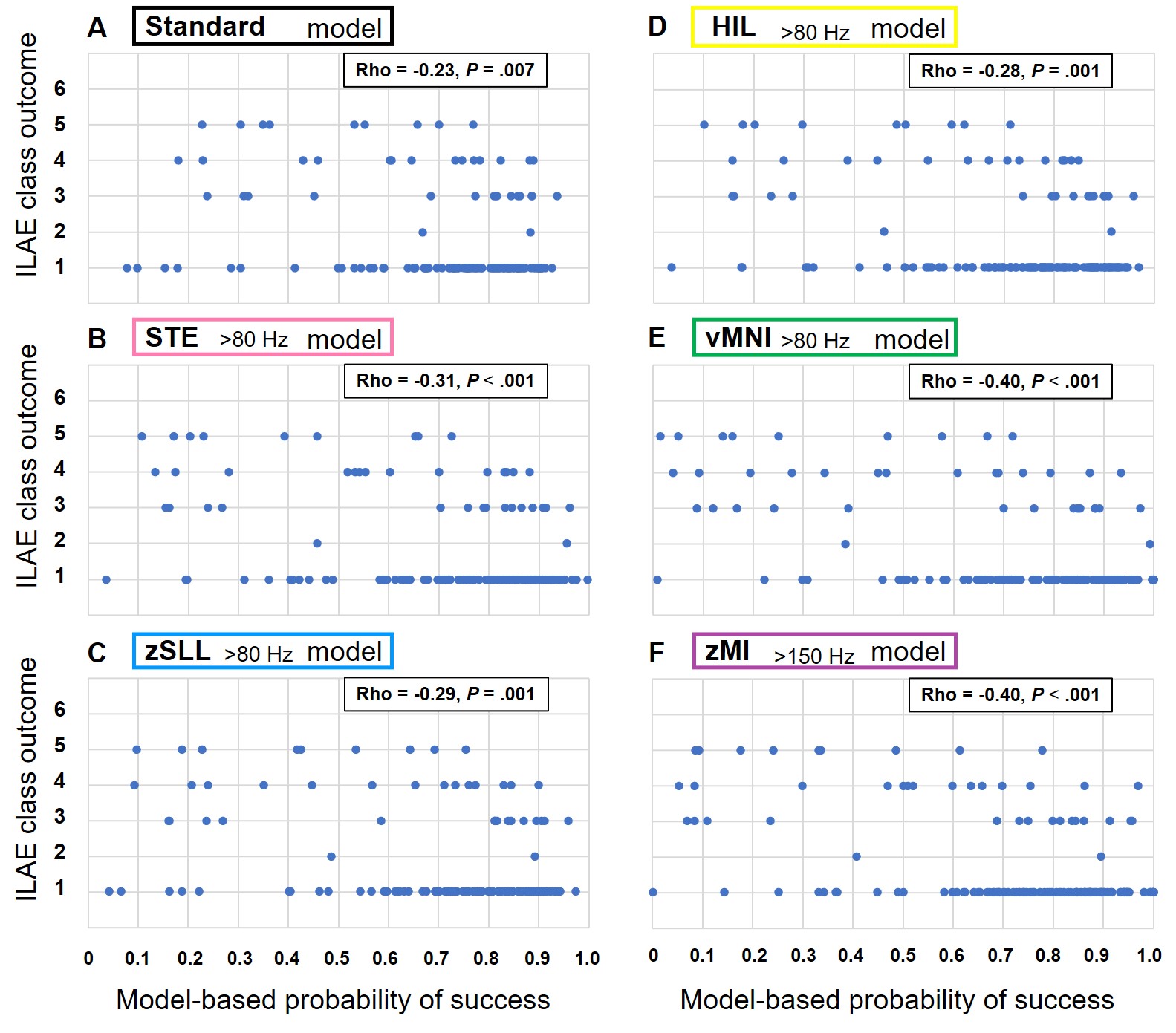


**Supplementary Figure 6: Correlation between model-based success probability and the ILAE outcome scale.** X-axis: Model-based probability of surgical success for a given patient; each model was cross-validated by the leave-one-out procedure. Y-axis: Postoperative seizure outcome according to the International League Against Epilepsy (ILAE) outcome classification. (A) Standard model. (B) STE_>80 Hz_ model. (C) zSLL_>80 Hz_ model. (D) HIL_>80 Hz_ model. (E) vMNI_>80 Hz_ model. (F) zMI_>150 Hz_ model. Increased model-based probability of success was associated with better postoperative seizure outcome in each model (Bonferroni-corrected *P* < .05 on the Spearman rank test).

**Supplementary Table 1:** **Outcome classification by the standard model.**

|  | *P* | OR | 95% C.I. |
| --- | --- | --- | --- |
| Age | .73 | 1.01 | 0.95-1.08 |
| Sex (1: male, 0: female) | .37 | 0.68 | 0.29-1.58 |
| Daily seizure | >.99 | 1.00 | 0.38-2.64 |
| The number of AEDs | **.037** | 0.56 | 0.33-0.97 |
| Hemisphere (1: left, 0: right) | .57 | 0.78 | 0.33-1.84 |
| MRI lesion | .50 | 1.35 | 0.57-3.21 |
| Habitual seizure event during iEEG | .27 | 2.00 | 0.58-6.95 |
| Incomplete resection of SOZ | **.002** | 0.15 | 0.05-0.51 |
| Resection of extratemporal region | .76 | 0.86 | 0.32-2.29 |
| Size of resection | .35 | 0.99 | 0.97-1.01 |

AEDs: antiepileptic drugs taken immediately before the electrode placement. C.I.: confidence interval. iEEG: intracranial EEG. OR: odds ratio. *P*: *P*-value. SOZ: seizure onset zone. *P* < .05 indicates significance in **bold** typeface. A larger number of AEDs and incomplete resection of the SOZ reduced the odds of surgical success.
